# Supplementary material for: Potential Transcriptional Enhancers in Coronaviruses: From Infectious Bronchitis Virus to SARS-CoV-2
Source: Int J Mol Sci. 2024 Jul 23;25(15):8012. doi: 10.3390/ijms25158012 (PMC11311688; doi:10.3390/ijms25158012)
Supplement: Supplementary file 1 [file ijms-25-08012-s001.zip › ijms-3100133-supplementary.pdf]

Supplement S1. Variation in IBV NSP16 extended duplex

NSP16 **N-terminus duplex (blue and red complementary halves) and**

		Duplex	ΔG	#	
1	UUAUAAUAUGCCUGAACUUUAUAAAGUUCAGAAUUGUGUUAUGG		-13.3	209	Chicken
2	UUAUAACAUGCCUGAACUUUAUAAAGUUCAGAAUUGUGUUAUGG	No	-15.2	4	
3	UUAUAACAUGCCUGAACUUUAUAAAGUUCAGAACUGUGUUAUGG	No	-15.2	3	
4	UUAUAACAUGCCUGAACUUUAUAAAGUUCAGACUUGUGUUAUGG	No	-15.2	1	
5	UUAUAAUAUGCCUGAGCUUUAUAAAGUUCAGAAUUGUGUUAUGG	No	-13.3	5	
6	UUAUAAUAUGCCUGAACUUUAUAAAGUUCAGAACUGUGUUAUGG	No	-13.3	95	
7	UUAUAAUAUGCCUGAACUUUAUAAAGUUCAGGACUGUGUUAUGG	No	-17.0	1	
8	UUAUAACAUGCCUGAACUUUAUAAAGUUCAUAACUGUGUUAUGG	No	-11.8	1	
9	UUAUAAUAUG-CUGAACUUUAUAAAGUUCAGAAUUGUGUUAUGG	No	-12.7	1	
10	UUAUAAUAUGCCUGAACUUUAUAAAGUUCAGAAUCGUGUUAUGG	No	-15.5	1	
11	UUAUAAUAUGCCUGAACUUUAUAAAGUUCAGAAUUGUAUUAUGG	No	-13.3	1	
12	UUAUAAUAUGCCUGAACUUUAUAAGGUUCAGAAUUGUGUUAUGG	No	-13.1	2	
13	UUAUAGUAUGCCUGAACUUUAUAAAGUUCAGAAUUGUGUUAUGG	No	-10.6	1	
14	UUACAAUAUGCCUGAACUUUAUAAAGUUCAGAAUUGUGUUAUGG	Yes	-11.0	4	
15	UUACAAUAUGCCUGAACUUUAUAAAGUCCAGAAUUGUGUUAUGG	Yes	-6.6	1	
16	UUACAAUAUGCCUGAACUUUACAAAGUCCAGAAUUGUGUUAUGG	Yes	-6.6	1	
17	UUAUAAUAUGCCUGAACUUUAUAAAGUUCAGAAUUGUGUUAUCU	Yes	-11.7	2	Pigeon
18	UUACAAUAUGCCUGAACUUUAUAAAGUUCAGAAUUGUGUUAUCU	Yes	-11.0	4	Pigeon
19	UUAUAAUAUGCCUGAACUUUACAAAGUUCAGAAUUGUGUUAUCU	Yes	-11.7	1	Chicken
20	UUAUAUUAUGCCUGAACUUUAUAAAGUUCAGAAUUGUGUUAUGG	Yes	-10.2	2	
21	UUAUAAUAUGCCCGAACUUUAUAAAGUUCAGAAUUGUGUUAUGG	Yes	-8.2	10	
22	UUAUAAUAUGCCUGAACUCUAUAAAGUUCAGAAUUGUGUUAUGG	Yes	-12.5	57	
23	UUAUAAUAUGCCUGAACUUUACAAAGUUCAGAAUUGUGUUAUGG	Yes	-13.3	14	
24	UUAUAAUAUGCCUGAACUUUAUAAAAUUCAGAAUUGUGUUAUGG	Yes	-7.8	1	
25	UUAUAAUAUGCCUGAACUUUAUAAAGUCCAGAAUUGUGUUAUGG	Yes	-8.8	30	
26	UUAUAAUAUGCCUGAACUUUAUAAAGUCCAGAAUUGUAUUAUGG	Yes	-8.8	13	
27	UUAUAAUAUGCCUGAACUUUAUAAAGUCCAGAAUUGCGUUAUGG	Yes	-7.0	2	
28	UUAUAAUAUGCCUGAACUUUAUAAAGUCCAGUAUUGUGUUAUGG	Yes	-8.9	1	
29	UUAUAAUAUGCCCGAACUUUAUAAAGUCCAGAAUUGUGUUAUGG	Yes	-4.1	1	
30	UUAUAAUAUGCCUGAACUUUACAAAGUCCAGAAUUGUGUUAUGG	Yes	-8.8	3	
31	UUAUAAUAUGCCUGAACUUUACAAAGUUCAGAACUGUGUUAUGG	Yes	-13.3	1	
32	UUAUAAUAUGCCUGAACUUUACAAAAUUCAGAAUUGUGUUAUGG	Yes	-7.8	2	
33	UUAUAAUAUGCCUGAGCUCUAUAAAGUUCAGAAUUGUGUUAUGG	Yes	-6.9	65	
34	UUAUAAUAUGCCUGAGCUUUAUAAAGUCCAGAAUUGUGUUAUGG	Yes	-9.1	1	
35	UUAUAAUAUGCCUGAGCUUUAUAAAGUUCAGAAUUGUAUUAUGG	Yes	-13.3	2	
36	UUAUAAUAUGCCUGAACUCUAUAAAGUCCAGAAUUGUGUUAUGG	Yes	-7.4	145	
37	UUAUAAUAUGCCUGAACUCUAUAAAGUCCAGAGUUGUGUUAUGG	Yes	-9.7	5	
38	UUAUAAUAUGCCUGAACUCUAUAAAGUUCAGAACUGUGUUAUGG	Yes	-12.5	6	
39	UUAUAAUAUGCCUGAACUCUACAAAGUUCAGAAUUGUGUUAUGG	Yes	-12.5	7	
40	UUAUAAUAUGCCUGAACUUUAUAAAGUACAGAAUUGUGUUAUGG	Yes	-8.8	1	
41	UUAUAAUAUGCCUGAACUUUAUAAAGUUCAGAAUUGCGUUAUGG	Yes	-11.4	2	
42	UUAUAAUUUGCCUGAACUUUAUAAAGUUCAGACUUGUGUUAUGG	Yes	-10.5	4	Duck
43	UUAUAACAUGCCUGAACUCUAUAAAGUUCAGAACUGUGUUAUGG	Yes	-14.4	8	
44	UUAUAACAUGCCUGAACUUUAUAAAGUCCAGAACUGUGUUAUGG	Yes	-10.7	1	
45	UUAUAAUUUGCCUGAACUUUAUAAAGUCCAGACUUGUGUUAUGG	Yes	-6.1	2	Duck
46	UUAUAAUAUGCCUGAACUCUAUAAAGUCCAGCAUUGUGUUAUGG	Yes	-8.7	1	
47	UUACAAUAUGCCUGAGCUCUAUAAAGUUCAGAAUUGUGUUAUGG	Yes	-10.2	1	
48	UUAUAAUAUGCGUGAGCUCUAUAAAGUUCAGAAUUGUGUUAUGG	Yes	-10.0	1	
49	UUAUAAUACGCCUGAGCUCUAUAAAGUUCAGAAUUGUGUUAUGG	Yes	-14.8	1	
50	UUAUAAUAUGCCUGAACUUUAUAAAGUCCAGAACUGUAUUAUGG	Yes	-8.8	1	
51	UUAUGUUAUGCCUGAACUUUAUAAAGUUCAGAACUGUGUUAUGG	Yes	-9.9	1	
52	UUAUAACAUGCCUGAACUAUAUAAAGUUCAGAAUUGCGUUAUGG	Yes	-11.7	1	
53	UUAUAAUAUGCCUGAGCUCUAUAAAGUUCAGAAUUGCGUUAUGG	Yes	-10.6	1	Turkey
54	UUAUAAUAUGCCUGAACUCUAUAAGGUUCAGAACUGUGUUAUGG	Yes	-12.6	3	Turkey
55	UUAUAAUAUGCCUGAACUCUACAAGGUUCAGAAUUGUGUUAUGG	Yes	-16.6	1	
56	UUAUAAUAUGCCUGA-CUCUACAAAGUUCAGAAUUGUGUUAUGG	Yes	-8.4	1	
57	UUACAAUAUGCCUGAAUUAUAUAAAGUUCAGAAUUGUAUUAUGG	Yes	-8.5	1	
58	UUAUAAUUUGCCUGAACUCUAUAAAGUUCAGACUUGCGUUAUGG	Yes	-10.2	1	Duck
59	UUAUACAAUGCCUGAGCUUUAUAAAGUUCAAGAUUGUGUUAUGG	Yes	-11.1	2	Goose, Swan
60	UUAUACAAUGCCUGAGCUCUAUAAAGUUCAAGAUUGUGUUAUGG	Yes	-10.3	1	Swan

This section details the animal source, geographical location, year of collection, and attenuation status when known of the IBV strains in GenBank used to analyze possible associations between mutations in the *NSP16* duplex and viral attenuation status. The strain GenBank accession numbers are shown in bold and highlighted in grey. A wild-type designation is highlighted in magenta, a pathogenic phenotype is highlighted in yellow, and vaccine/vaccine-derived/attenuated strains are highlighted in red. Groups 1 and 36 represent the peaks of the two clusters when strains are organized by the minimum free energy of the *NSP16* duplex. Visual inspection of these two groups shows scarce red highlights in group 1 in contrast to their preponderance in group 36.

No mutations relative reference strain (NC_001451.1)[1]: **GQ504724.1** (Chicken, Massachusetts/USA, strain Massachusetts, 1941, wild-type); **MK990808.1** (*Gallus gallus*, Australia, 1962; genotype: GI-5); **FJ904720.1** (Chicken, Massachusetts/USA, 1965; genotype: GI-5; McKinley et al., 2011, pathogenic, Liu et al. 2013); **FJ904721.1** (Chicken, Massachusetts/USA, 1972; McKinley et al., 2011); **MK990813.1** (*Gallus gallus*, Australia, 1973, genotype: GI-6); **FJ904722.1** (Chicken, Massachusetts/USA, 1985, strain: Mass41 1979, McKinnley et al., 2011); **FJ904723.1** (Chicken, Massachusetts/USA, 1985, strain: Mass41 1985, McKinnley et al. 2011); **MK982928.1** (*Gallus gallus*, Australia, 1988, genotype: GI-5); **MK990812.1** (*Gallus gallus*, Australia, 1990, genotype: GI-6); **LC716900.1** (*Gallus gallus domesticus*, Japan, 1993); **MN128088.1** (Chicken, Taiwan, 1995, 3rd passage, strain: TW2296/95w [wild-type strain of MN128086.1], Tsai et al., 2020); **MN128086.1** (Chicken, Taiwan, 1995, 79th passage, strain: TW2296/95v [vaccine strain of MN128088.1], Tsai et al., 2020); **MK972912.1** (*Gallus gallus*, Australia, 1999, genotype: GI-6); **MK982928.1** (*Gallus gallus*, Australia, 1999, genotype: GI-5); **MK957244.1** (*Gallus gallus*, Brazil, 2000); **AJ311317.1** (IBV, United Kingdom, 05/15/2001, strain: Beaudette CK); **MK990809.1** (*Gallus gallus*, Australia, 2002, genotype: GI-5); **M94356.1** (IBV, 02/08/2002, strain: Beaudette M42); **EU714029.1** (*Gallus gallus*, China, 11/2002, responsible for chick outbreak of nephritis); **KU556806.1** (*Gallus gallus*, Australia, 02/01/2003, isolate Ck/Aus/NI/03, Quinteros et al., 2016); **AY851295.1** (Chicken, USA/Massachusetts, 12/09/2004, strain: Mass 41); **HM449163.1** (Chicken, China, 12/28/2004, strain: Jin-13, respiratory type); **AY692454.1** (Beaudette-Vero-cell adapted, 07/12/2004); **DQ001338.1** (Vero cell, 04/07/2005, strain: Beaudette, chicken embryo cell passage 3); **DQ001339.1** (Vero cell passage 65, 04/07/2005); **DQ288927.1** (Chicken, China, 10/26/2005, nephrogenic strain); **JF893452.2** (Chicken, China, 2005, isolate YN, nephrogenic strain); **OM525802.1** (Broiler chicken, China, 2005, isolate D532/9/CH/05); **MH181793.1** (Chicken, China, 2006, strain HH06); **KX252779.1** (*Gallus gallus*, China, 2006, strain ck/CH/LLN/06I, multiple organ tropisms); **OM525806.1** (Broiler chicken, Slovakia, 2006, isolate D722/SK/06); **KX219792.1** (*Gallus gallus*, China, 05/2006, islate ck/CH/LGS/06I genotype: LX4, multiorgan tropism); **DQ834384.1** (Chicken, China, 06/30/2006, strain M41, serotype: Massachusetts [vaccine strain]); **KX375805.1** (*Gallus gallus*, China, 2007, isolate ck/CH/LJS/07V, genotype: LX4); **KX252782.1** (*Gallus gallus*, China, 2007, strain ck/CH/LLN/07I, multiporgan tropism); **HM245923.1** (Chicken, China, 2007, isolate DY07); **MK990810.1** (*Gallus gallus*, Australia, 2007, strain Ck/Aus/V1/07,

genotype: GV-1); KX219791 (*Gallus gallus*, China, 02/2007, isolate ck/CH/LHLJ/07I, genotype: LX4, multiorgan tropism, pathogenic, Liu et al. 2013); KF663560.1 (Chicken, China, 11/2007, isolate ck/CH/IBWF/2007, multiorgan tropism); KU900738.1 (*Gallus gallus*, South Korea, 2008, strain SNU-8065); KX364296.1 (*Gallus gallus*, China, 2008, strain ck/CH/LJL/08-9, genotype: LX4); KX275394.1 (*Gallus gallus*, China, 2008, strain ck/CH/LSD08-8, genotype: LX4); KX302860.1 (*Gallus gallus*, China, 2008, strain ck/CH/LSD08-7, genotype: LX4); KX236005.1 (*Gallus gallus*, China, 2008, strain ck/CH/LJL/08-1); KX252788.1 (*Gallus gallus*, China, 2008, strain ck/CH/LHLJ/08-6, geonotype: LX4); KM586818.1 (Chicken, China, 08/2008, isoate P100, genotype: QX-type); FJ807653.1 (Chicken, China, 2008, isolate chicken/JS/YZ07/2008); KU556807.1 (*Gallus gallus*, Australia, 2008, isolate Ck/Aus/N1/08); EU637854.1 (Chicken, China, 04/11/2008, strain CK/CH/LSD/05I); KF853202.1 (Chicken, China, 07/11/2008, isolate SDZB0808, genotype: QX-type); MN517816.1 (*Gallus gallus*, China, 2009, strain cK/CH/LJL/090608); KX259249.1 (*Gallus gallus*, China, 2009, isolate ck/CH/LJL/090330, nephropathogenic strain with varying levels of virulence in specific pathogen-free chickens); KX259254.1 (*Gallus gallus*, China, 2009, isolate ck/CH/LSD/090314, pathogenic); KX275392.1 (*Gallus gallus*, China, 2009, isolate ck/CH/LHLJ/110943, genotype LX4, pathogenic); KX302864.1 (*Gallus gallus*, China, 2009, strain ck/CH/LSD/09091, geotype: LX4, pathogenic); KX236012.1 (*Gallus gallus*, China, 2009, isolate ck/CH/LSD/091003, pathogenic); KX252789.1 (*Gallus gallus*, China, 2009, isolate ck/CH/LHLJ/090605, pathogenic); KX252783.1 (*Gallus gallus*, China, 2009, isolate ck/CH/LHLJ/090510, pathogenic); KX252784.1 (*Gallus gallus*, China, 2009, isolate ck/CH/LLN/090910, pathogenic); KX252790.1 (*Gallus gallus*, China, 05/2009, isolate ck/CH/LHLJ/090515, genotype LX4, pathogenic); KX302869.1 (*Gallus gallus*, China, 08/2009, isolate ck/CH/LHLJ/090806, genotype LX4, pathogenic); KX236016.1 (*Gallus gallus*, China, 09/2009, isolate ck/CH/LHB/090921, genotype LX4, pathogenic); JF732903.1 (Chicken, China, 04/2009, isolate Sczy-3, pathogenic); KF411041.1 (Chicken, China, 11/10/2009, CK/CH/LGX/091109, pathogenic); KJ425504.1 (*Gallus gallus*, China, 12/05/2009, isolate ck/CH/LHLJ/091205); KX236006.1 (*Gallus gallus*, China, 2010, isolate ck/CH/LSD/100305); KU361187.1 (Chicken, China, 2010, isolate CK/CH/2010/JT-1, originated by recombination of various strains, pathogenic); JF330898.1 (Chicken, China, 2010, isolate ck/CH/LHB/100801); JX840411.1 (Chicken, China, 10/2010, strain YX10); OR050557.1 (Layer, South Korea, 12/02/2010, isolate IBV/Korea/415/2010); KX236011.1 (*Gallus gallus*, China, 09/2012, isolate ck/CH/LSD/091203); KX364290.1 (*Gallus gallus*, China, 2010, isolate ck/CH/LJL/101150); KX259248.1 (*Gallus gallus*, China, 2010, isolate ck/CH/LHN/101211); KX252785.1 (*Gallus gallus*, China, 2010, isolate ck/CH/LSD/100311); MN531555.1 (*Gallus gallus*, China, 2011, isolate cK/CH/LSD/1112145); MN509589 (*Gallus gallus*, China, 2011, isolate cK/CH/LHLJ/110664); KX364292.1 (*Gallus gallus*, China, 2011, isolate ck/CH/LSD/110739, genotype LX4); KX400753.1 (*Gallus gallus*, China, 2011, isolate ck/CH/LHB/110123, genotype LX4); MK032181.1 (*Gallus*, China, 2011, isolate gammaCoV/ck/China/I0347/11); KX236000.1 (*Gallus gallus*, China, 2011, isolate ck/CH/LSD/110912, genotype LX4); KX247127.1 (*Gallus gallus*, China, 2011, isolate ck/CH/LHB/111190); KY721498.1 (Chicken, China, 2011); KC136209.1 (Chicken, China, 2011, isolate ck/CH/LJL/110302); KU317090.1 (*Gallus gallus*, China, 2011, strain SAIBK2, genotype LX4, nephropathogenic, Wu X et al. 2016); KU900742.1 (*Gallus gallus*, South Korea, 2011); KX375806.1 (*Gallus gallus*, China, 2011, isolate ck/CH/LHLJ/110673, genotype LX4, chimeric

virus with an LX4-like backbone, except for the S gene, which might be from an unknown strain); **KX375807.1** (*Gallus gallus*, China, 2011, isolate ck/CH/LHLJ/110836, genotype LX4); **KF460437.1** (*Gallus gallus*, Australia, 2011, isolate VicS-v, serotype; sub-group 1); **MT984595.1** (*Gallus gallus*, Romania, 2011); **MT984590.1** (*Gallus gallus*, Hungary, 2011, isolate D1623/1/1/2011/HU); **OR001749.1** (Korean native chicken, South Korea, 07/25/2011, strain IBV/Korea/117/2011); **KX259255.1** (*Gallus gallus*, China, 11/2011, strain ck/CH/LJS/111111, genotype LX4); **KX219793.1** (*Gallus gallus*, China, 11/2011, strain ck/CH/LHB/111168, genotype: LX-4); **OR050546.1** (Broiler, South Korea, 04/11/2011, isolate IBV/Korea/55/2011); **KX219796.1** (*Gallus gallus*, China, 12/2011, strain ck/CH/LXJ/111265, genotype LX4); **MN531554.1** (*Gallus gallus*, China, 2012, strain cK/CH/LLN/120611); **KX372249.1** (*Gallus gallus*, China, 2012, strain ck/CH/LHLJ/121219, genotype LX4); **MT984602.1** (*Gallus gallus*, Romania, 2012, strain D20222/2/3/2012/RO); **MK728875.1** (*Gallus gallus*, United Kingdom, 02/2012, known pathogenic *in vivo* phenotype); **MG448607.1** (*Gallus gallus*, China, 2012, isolate ck/CH/LHB/121042); **KX389094.1** (*Gallus gallus*, China, 2012, strain ck/CH/LBJ/120481, genotype LX4); **KX236013.1** (*Gallus gallus*, China, 2012, strain ck/CH/LSD/120742); **KX236014.1** (*Gallus gallus*, China, 2012, strain ck/CH/LHB/120497); **KF574761.1** (Chicken, China, 2012, isolate SDIB821/2012); **KX219801.1** (*Gallus gallus*, China, 04/2012, strain ck/CH/LSD/120437, genotype LX4); **KX252786.1** (*Gallus gallus*, China, 06/2012, strain ck/CH/LLN/120605, genotype LX4); **KU556804.1** (*Gallus gallus*, Australia, 05/07/2012, isolate Ck/Aus/N1/88, subgenotype 2, pathogenic, isolated from previously vaccinated chickens, Quinteros et al 2016); **KU556805.1** (*Gallus gallus*, Australia, 05/07/2012, islate Armidale A3); **KX259257.1** (*Gallus gallus*, China, 08/2012, strain ck/CH/LJS/120848, genotype LX4); **KF931628.1** (*Gallus gallus*, Australia, 08/06/2012, isolate Vics-del); **KX219798** (*Gallus gallus*, China, 09/2012, isolate ck/CH/LSD/120913); **KX348115.1** (*Gallus gallus*, China, 09/2012, isolate ck/CH/LAH/120907); **KJ128295.1** (Chicken, China, 12/20/2012, isolate CK/CH/SD/121220); **MK972911.1** (*Gallus gallus*, Australia, 2013, isolate Ck/Aus/Q1/13); **MH508703.1** (Chicken, China, 01/01/2013); **KY421673.1** (Chicken, China, 01/01/2013, isolate SD, genotype QX-like, pathogenic); **KX434788.1** (*Gallus gallus*, China, 2013, isolate ck/CH/LHLJ/130622, genotye LX4); **KX372250.1** (*Gallus gallus*, China, 2013, isolate ck/CH/LSD/130205, genotype LX4); **KX302874.1** (*Gallus gallus*, China, 2013, isolate ck/CH/LGS/131148, genotype LX4); **KX302871.1** (*Gallus gallus*, China, 2013, strain ck/CH/LHuB/131123, genotype LX4); **KX302868.1** (*Gallus gallus*, China, 2013, strain ck/CH/LHLJ/130744, genotype LX4); **KX236008.1** (*Gallus gallus*, China, 2013, isolate ck/CH/LDL/130325); **KX252774.1** (*Gallus gallus*, China, 2013, strain ck/CH/LHB/130589); **KX252775.1** (*Gallus gallus*, China, 2013, strain ck/CH/LHB/130628); **KX185057.1** (*Gallus gallus*, China, 2013, strain ck/CH/LHLJ/95I); **KP036505.1** (*Gallus gallus*, China, 2013, strain ck/CH/LJL/130925); **KX219799.1** (*Gallus gallus*, China, 02/2013, strain ck/CH/LSD/130211, genotype LX4); **KX247129.1** (*Gallus gallus*, China, 03/2013 strain ck/CH/LHB/130337, genotype LX4); **KX247128.1** (*Gallus gallus*, China, 05/2013, strain ck/CH/LHB/130569, genotype LX4); **KX219800.1** (*Gallus gallus*, China, 06/2013, strain ck/CH/LSD/130611, genotype LX4); **KY805846.1** (*Gallus gallus*, Egypt, 2014, strain IBV/Ck/EG/CU/4/2014, genotype GI-23 lineage, Abozeid et al. 2017); **KX364294.1** (*Gallus gallus*, China, 2014, strain ck/CH/LBJ/140528, genotype LX4); **KX425847.1** (*Gallus gallus*, China, 2014, strain ck/CH/LJL/140734, genotype LX4); **KX275391.1** (*Gallus gallus*, China, 2014, strain ck/CH/LHLJ/140927); **KX302873.1** (*Gallus gallus*, China, 2014

strain ck/CH/LJL/140924, genotype LX4); **KX185058.1** (*Gallus gallus*, China, 2014, strain ck/CH/LGS/08I); **KP790145.1** (*Gallus gallus*, China, 2014, strain CK/CH/LHLJ/141105); **KP790146.1** (*Gallus gallus*, China, 2014, strain CK/CH/LHLJ/140901); **KX252773.1** (*Gallus gallus*, China, 05/2014, strain ck/CH/LHB/140542, genotype LX4); **KX275390.1** (*Gallus gallus*, China, 11/2014, strain ck/CH/LHLJ/141103, genotype LX4); **KX302875.1** (*Gallus gallus*, China, 11/2014, strain ck/CH/LJL/100512, genotype LX4); **KX302867.1** (*Gallus gallus*, China, 2015, strain ck/CH/LJL/150430, genotype LX4); **ON260867.1** (*Gallus gallus*, China, 02/21/2015, strain CK/CH/SD/MH20 2015, genotype GI-19); **KX219795.1** (*Gallus gallus*, China, 03/2015, strain ck/CH/LSD/150311, genotype LX4); **KY620116.1** (*Gallus gallus*, China, 2016, strain gammaCoV/ck/China/I1101/16, genotype LX4); **MH539771.1** (*Gallus gallus*, Canada, 2015/2016, isolate IBV_SES_15AB-01, serotype: Massachusetts, pathogenic, Amarasinghe et al. 2018); **MH539772.1** (*Gallus gallus*, Canada, 2015/2016, isolate IBV_SES_15SK-02, similar to MH539772.1 but less pathogenic); **OR050560.1** (Broiler, South Korea, 04/21/2016, isolate IBV/Korea/85/2016); **OR050552.1** (Broiler, South Korea, 04/29/2016, isolate IBV/Korea/87/2016); **OR050563.1** (Broiler, South Korea, 05/07/2016, isolate IBV/Korea/111/2020); **MW222189.1** (Chicken, China, 12/11/2016, isolate GX-NN160421, 3-nt deletion in N gene, low virulence but prolonged virus shedding, Lv Di et al 2021); **LC710779.1** (*Gallus gallus*, Germany, 2016, isolate Beaudette-FUB, genetic engineering constructs); **OR050545.1** (Broiler, South Korea, 04/12/2017, isolate IBV/Korea/37/2017); **OR050550.1** (Broiler, South Korea, 05/23/2017, isolate IBV/Korea/76/2017); **MK329221.1** (Chicken, Taiwan, 07/05/2017, isolate CK-CH-GX-YL17, genotype TC07-2); **MK309398.1** (Chicken, China, 07/05/2017, isolate CK/CH/GD/HY16, genotype TC07-2); **MH020185.1** (Chicken, China, 10/18/2017, isolate CK/CH/HD/171018, genotype QX-like); **MK032177.1** (*Gallus*, China, 2017, isolate gammaCoV/ck/China/I0724/17); **MK032178.1** (*Gallus*, China, 2017, isolate gammaCoV/ck/China/I0718/17); **MK032179.1** (*Gallus*, China, 2017, isolate gammaCoV/ck/China/I0722/17); **MK032180.1** (*Gallus*, China, 2017, strain gammaCoV/ck/China/I0737/17); **KY799582.1** (*Gallus gallus*, China, 03/20/2017, strain ck/CH/LSC/99I, pathogenic, Jian L et al 2018); **MN199463.1** (*Gallus gallus*, South Korea, 04/05/2017, isolate R17/19); **MN199464.1** (*Gallus gallus*, South Korea, 05/04/2017, isolate R17/27); **MN199465.1** (*Gallus gallus*, South Korea, 05/19/2017, isolate R17/36); **MK423876.1** (Pheasant, China, 2017); **MK423877.1** (Pheasant, China, 2017); **MK644086.1** (Chicken, China, 2018, E160_YN, genotype GI-22, attenuated, suggested secondary to S and 5a, Zhao Y et al 2019); **OP868815.1** (Chicken, China, 2018, isolate China/Sichuan/QX-like/SczyC30/2020, genotype QX); **OR050543.1** (Broiler, South Korea, 02/08/2018, isolate IBV/Korea/17/2018); **MW896950.1** (Chicken, China, 04/01/2018, isolate s23-194345); **MN199466.1** (*Gallus gallus*, South Korea, 04/17/2018 isolate R18/23); **MW351625.1** (Chicken, China, 2019, isolate IBV-rSD-H238A); **MW351623.1** (Chicken, China, 2019, isolate IBV-rSD, genotype GX-like); **MW351624.1** (Chicken, China, 2019, isolate IBV-rSD-H223A, genotype QX-like, endoU deficient mutant); **MW351626.1** (Chicken, China, 2019, isolate IBV-rSD-K278A, engineered endoribonuclease deficient mutant); **MW351628.1** (Chicken, China, 2019, islate IBV-rSD-Y334A, endoU deficient mutant); **MW351629.1** (Chicken, China, 2019, islate IBV-rSD-H223A/H238A/K278A/Y334A, endoU deficient mutant); **MW815494.1** (Avian, China, 04/2019, isolate CK/CH/TJ1904); **ON310377.1** (*Gallus gallus*, China, 05/2019, isolate CH/LN/2019, genotype QX-like); **MK937830.1** (*Gallus*, China, 05/15/2019, isolate M41); **MN894506.1** (*Gallus gallus*, China, 07/16/2019, isolate

CK/CH/HD/190716, genotype QX-like); **OM525799.1** (Layer chicken, Ohio, United States, 2020, isolate DMV25/OH/20, da Silva et al. 2022); **OM525800.1** (Layer chicken, Ohio, United States, 2020, isolate DMV26/OH/20, da Silva et al. 2022); **OM525801.1** (Layer chicken, Ohio, United States, 2020, isolate DMV28/OH/20, da Silva et al. 2022); **MW222181.1** (*Gallus gallus*, China, 2020, isolate IBV/M41/Y191); **MW419300.1** (*Gallus gallus*, China, 2020, strain IBV/M41/Y83); **MW248465.1** (*Gallus gallus*, China, 2020, isolate IBV/M41/Y82); **MW429059.1** (*Gallus gallus*, China, 2020, isolate IBV/M41/Y164); **MW429060.1** (*Gallus gallus*, China, 2020, isolate IBV/M41/Y195); **MW429062.1** (*Gallus gallus*, China, 2020, isolate IBV/M41/N56); **MW791835.1** (Chicken, China, 2020, isolate CK/CH/FJ/202005, genotype VI, 10 day old SPF chicken embryo); **MT460496.1** (Chicken, China, 05/03/2020, isolate CK/CH/LAH/1806); **MW436705.1** (*Gallus gallus*, China, 2020, isolate IBV/M41/Y25); **MW446887.1** (*Gallus gallus*, China, 2020, isolate IBV/M41/Y192); **MW446888.1** (*Gallus gallus*, China, 2020, isolate IBV/M41/Y27); **MW792511.1** (*Gallus gallus*, China, 2020, strain I0302/20); **MW792514.1** (*Gallus gallus*, China, 2020, strain I0303/20); **MZ368698.1** (*Gallus gallus*, Brazil, 07/17/2020, strain Beaudette, genotype GI-1); **OP737825.1** (Chicken, China, 04/20/2021, isolate IBV/chicken/Jiangsu/J1231/2021, pathogenic); **OP737826.1** (Chicken, China, 04/20/2021, isolate IBV/chicken/Jiangsu/J1237/2021, pathogenic); **MW847253.1** (*Gallus gallus*, Germany, 03/31/2021, artificial chromosome with Beaudette strain); **ON951674.1** (Chicken, China, 11/18/2021, strain CK/CH/SX/MJ4); **ON951675.1** (Chicken, China, 11/18/2021, strain CK/CH/SX/MJ5); **ON951676.1** (Chicken, China, 11/18/2021, strain CK/CH/SX/MJ9); **OQ716703.1** (*Gallus gallus*, Indonesia, 12/01/2021, isolate IBV/ID761/2021, genotype QX-like); **OM912707.1** (Chicken, USA, 03/02/2022, isolate IBV/ck/MEX/2960/21); **OQ716701.1** (*Gallus gallus*, Indonesia, 06/03/2022, isolate IBV/ID865/2022, genotype QX-like).

One mutation (U to C at position 19,532 of reference strain NC_001451.1, without effect on extended duplex)[2]: **KX258195.1** (*Gallus gallus*, Brazil, 2013, chicken embryonated eggs 3rd passage); **MG913342.1** (*Gallus gallus*, Brazil, 2013, isolate AvCoV/Gallus gallus/Brazil/sample 38/2013 GI-11, 3rd passage in chicken embryos); **ON41989.1** (*Gallus gallus*, Argentina, 2013); **KY626044.1** (*Gallus gallus*, Brazil, 2016, strain BR-I, genotype: GI-11)

Two mutations (U to C at positions 19,532 and 19,559 of reference strain NC_001451.1, without effect on extended duplex)[3]: **MK990811.1** (*Gallus gallus*, Australia, 1971, Ck/Aus/V1/71, genotype: GI-6); **MT701511.1** (*Gallus gallus*, China, 09/2017, isolate GD17/04, genotype: 4/91 [GI-13]); **OP737824.1** (Chicken, China, 05/11/2021, IBV/chicken/Henan/H1036/2021)

Two mutations (U to C at positions 19,532 and 19,558 of reference strain NC_001451.1, without effect on extended duplex)[4]: **MK953937.1** (*Gallus gallus*, Brazil, 2014, strain Brazil/SP55)

One mutation (A to G at position 19,541 of reference strain NC_001451.1, without effect on extended duplex)[5]: **FN430415.1** (IBV, Nigeria, 2006, isolate NGA/A116E7/2006 pathogenic, Ducatez et al. 2009); **MZ325296.1** (*Gallus gallus*, Cameroon, 2013, strain D2326/3/13/CM); **MZ325297.1** (*Gallus gallus*, Cameroon, 2013, strain D2326/4/13/CM); **MK217373.1** (*Gallus*, China, 2017, strain I0725/17); **MW729513.1** (*Gallus*, China, 2020)

One mutation (U to C at position 19,559 of reference strain NC_001451.1, without effect on extended duplex)[6]: **HM245924.1** (Chicken, China, 2004, isolate CQ04-1, pathogenic); **MK581205.1** (*Gallus gallus*, Poland, 10/23/2004, strain gammaCoV/Ck/Poland/548/2004); **OM525803.1** (Layer chicken, France, 2005, isolate D535/4/FR/05, da Silva et al. 2022); **OM525804.1** (Chicken, Greece, 2005, isolate D591/2/GR/05, da Silva et al. 2022); **FN430414.1** (IBV, USA, 2005, isolate ITA/90254/2005); **OM525805.1** (Chicken, Hungary, 2006, isolate D683/HU/06, da Silva et al. 2022); **KX364303.1** (*Gallus gallus*, China, 2007); **HO850618.1** (*Gallus gallus*, China, 08/25/2007); **MN509587.1** (*Gallus gallus*, United Kingdom, 01/2009, strain cK/CH/LDL/091021); **KX302863.1** (*Gallus gallus*, China, 2009); **KT886454.1** (*Gallus gallus*, Poland, 2009); **KP118894.1** (*Gallus gallus*, China, 2009); **KX259251.1** (*Gallus gallus*, China, 2010); **KX302872.1 9** (*Gallus gallus*, China, 2010); **MT984583** (*Gallus gallus*, Hungary, 2010); **JQ088078.1** (Chicken, Sweden, 2010); **KF663561.1** (Chicken, China, 04/2011); **KX259252.1** (*Gallus gallus*, China, 2011); **KX434790.1** (*Gallus gallus*, China, 2011); KX640829.1 (*Gallus gallus*, China, 2011); **MN531556.1** (*Gallus gallus*, China, 2011); **KX364300.1** (*Gallus gallus*, China, 2011); **KP868572.1** (*Gallus gallus*, China, 2011); **MN548289.1** (*Gallus gallus*, United Kingdom, 01/2011); **MT984589.1** (*Gallus gallus*, Ukraine, 2011); **MT984591.1** (*Gallus gallus*, Romania, 2011); **MT984598.1** (*Gallus gallus*, Belarus, 2011); **KY421672.1** (Chicken, China, 2012); **MT984597.1** (*Gallus gallus*, Romania, 2012); **MT984598.1** (*Gallus gallus*, Portugal, 2012); **KF663559.1** (Chicken, China, 06/2012); **KC013541.1** (Chicken, China, 07/12/2012); **KC119407.1** (Chicken, China, 07/20/2012); **MT984599.1** (*Gallus gallus*, Greece, 2013); **KX302861.1** (*Gallus gallus*, China, 2013); **KX302865.1** (*Gallus gallus*, China, 2013); **OQ117368.1** (Chicken, China, 2013); **KY407556.1** (*Gallus gallus*, China, 2014); **KU361188.1** (Chicken, China, 2014); **KP790143.1** (*Gallus gallus*, China, 2014); **KP790144.1** (*Gallus gallus*, China, 2014); **KT946798.1** (Chicken, China, 01/01/2014); **KX364295.1** (*Gallus gallus*, China, 05/ 2014); **MG738154.1** (*Gallus gallus*, Malaysia, 2014); **MG738155** (*Gallus gallus*, Malaysia, 2015); **MG233398.1** (Chicken, Iran, 2015); **MH924835.1** (*Gallus*, China, 2016); **MT591566.1** (Chicken, Germany, 02/2016); **MG517474.1** (Chicken, China, 07/01/2016); **MG197727.1** (Chicken, China, 11/01/2016); **MN307884.1** (Yellow feather broiler, China, 2017); **MN217372.1** (*Gallus*, China, 2017); **KY933089.1** (Chicken, United Kingdom, 04/12/2017); **KY933090.1** (Chicken, United Kingdom, 04/12/2017); **MW896952.1** (Chicken, China, 04/01/2018); **ON260866.1** (*Gallus gallus*, China, 08/13/2018); **OR978261.1** (Chicken, Viet Nam, 2019); **OQ725698.1** (*Gallus gallus*, Tanzania, 05/03/2019); **MT978193.1** (*Gallus*, China, 2020); **MZ456995.1** (Chicken, China, 05/12/2020); **OK423447.1** (*Gallus sp.*, China, 2021); **OR840944.1** (Chicken, Viet Nam, 2021); **OM937935.1** (*Gallus*, China, 2022); **OM937936.1** (*Gallus*, China, 2022); **OM937937.1** (*Gallus*, China, 2022); **OM937938.1** (*Gallus*, China, 2022); **OM937939.1** (*Gallus*, China, 2022); OM937940.1 (*Gallus*, China, 2022); **OM937941.1** (*Gallus*, China, 2022); **OM937942.1** (*Gallus*, China, 2022); **OM937943.1** (*Gallus*, China, 2022); **OM937944.1** (*Gallus*, China, 2022); **OM953425.1** (*Gallus*, China, 2022); **OM988131.1** (*Gallus sp.*, China, 2022); **OM988132.1** (*Gallus sp.*, China, 2022); **OM988133.1** (*Gallus sp.*, China, 2022); **OM988135.1** (*Gallus sp.*, China, 2022); **OM988136.1** (*Gallus sp.*, China, 2022); **OM988137.1** (*Gallus sp.*, China, 2022); **OM988139.1** (*Gallus sp.*, China, 2022); **OM988140.1** (*Gallus sp.*, China, 2022); **OM988141.1** (*Gallus sp.*, China, 2022); **OM988142.1** (*Gallus sp.*, China, 2022); **OM988143.1** (*Gallus sp.*, China, 2022); **OM988144.1** (*Gallus sp.*, China, 2022); **OM988145.1** (*Gallus sp.*, China, 2022); **OM988147.1** (*Gallus sp.*, China, 2022); **OM988149.1** (*Gallus sp.*, China, 2022); **OM988151.1** (*Gallus sp.*, China, 2022); **OM988154.1** (*Gallus sp.*, China,

2022); **OM988155.1** (*Gallus sp.*, China, 2022); **OM988157.1** (*Gallus sp.*, China, 2022); **OM988158.1** (*Gallus sp.*, China, 2022); **ON951677.1** (Chicken, China, 11/18/2021); **OR208629.1** (Mallard, China, 06/2022)

Two mutations (A to G and U to C at positions 19,557 and 19,559, respectively of reference strain NC_001451.1, without effect on extended duplex)[7]: **OR791595.1** (*Gallus Gallus*, Netherlands, 2019, gCoV/ck/Netherlands/D2860//2019, genotype: GVIII)

Two mutations (U to C at positions 19,532 and 19,559, respectively, of reference strain NC_001451.1, without effect on extended duplex)[8]: **KX252791.1** (Gallus gallus, China, 1998, cK/CH/LLN/98I, genotype: LX4)

One deletion at position 19,536 of reference strain NC_001451.1, without effect on extended duplex)[9]: **KX348117.1** (*Gallus gallus*, China, 07/2012)

One mutation (U to C at position 19,560 of reference strain NC_001451.1, without effect on extended duplex)[10]: **EU022526.1** (Turkey, Indiana/USA, 07/08/2007, isolate TCoV-ATCC, embryonated turkey eggs [day 21])

One mutation (G to A at position 19,563 of reference strain NC_001451.1, without effect on extended duplex)[11]: **KX077987.1** (*Gallus gallus*, China, 2015, strain ck/CH/LDL/150434-III).

One mutation (A to G at position 19,550 of reference strain NC_001451.1, without effect on extended duplex)[12]: **MT984584** (Gallus gallus, Hungary, 2011, strain 211776/Imrehegy/2011/HU, Bali et al. 2021); **MT984600.1** (*Gallus gallus*, Poland, 2013, strain D2353/2013/PL, Bali et al. 2021).

One mutation (A to G at position 19,531 of reference strain NC_001451.1, without effect on extended duplex)[13]: **MW729512.1** (*Gallus*, China, 2020).

One mutation (U to C at position 19,529 of reference strain NC_001451.1, introducing mismatch in extended duplex)[14]: **LC716901.1** (*Gallus gallus domesticus*, Japan, 1998, strain JP/Shimane/98); **MN025323.1** (Pigeon, China, 12/01/2018, pigeon CoV dominant in China); **LC663496.1** (*Gallus gallus*, Japan, 12/03/2021, strain C-78E128, attenuated strain obtained by 128 egg-passages); **LC683780.1** (*Gallus gallus domesticus*, Japan, 02/10/2022, strain JP/Yamagata/2017).

Two mutations (U to C at positions 19,529 and 19,553 of reference strain NC_001451.1, introducing mismatches in extended duplex)[15]: **JQ977697.1** (*Gallus gallus*, South Korea, 2008, isolate SNU8067)

Three mutations (U to C at positions 19,529, 19547 and 19,553 of reference strain NC_001451.1, introducing mismatches in extended duplex)[16]: **KX266757.1** (*Gallus gallus*, Taiwan, 2008, isolate 3575/08, strong respiratory and renal pathogenicity, delayed expression of a subset of early

innate immune genes, Lin S-Y et al. 2016, isolated from broilers vaccinated with the attenuated viral vaccine derived from a Taiwan strain 2575/98)

Two mutations at the end (CU instead of GG introducing two mismatches in extended duplex) relative reference strain (NC_001451.1)[17]: KT254299.1 (Pigeon, China, 2014, strain PdCoV/PG/Guangdong/1507/2014, pigeon-dominant CoV); MN025324 (Pigeon, China, 12/01/2018, isolate PdCoV/PG/Hebei/15-4-6/2018, pigeon-dominant CoV).

Three mutations (CU instead of GG at the end and U to C at position 19,529 of reference strain NC_001451.1, introducing two mismatches in extended duplex) relative reference strain (NC_001451.1)[18]: KT254297.1 (Pigeon, China, 2014, isolate PdCoV/PG/Guangdong/1418/2014, pigeon-dominant CoV); KT254298.1 (Pigeon, China, 2014, isolate PdCoV/PG/Guangdong/1068/2014, pigeon-dominant CoV); MN025321.1 (Pigeon, China 12/01/2018, isolate PdCoV/PG/Jiangsu/9-1-3/2018, pigeon-dominant CoV); MN025322.1 (Pigeon, China 12/01/2018, isolate PdCoV/PG/Jiangsu/9-4-6/2018, pigeon-dominant CoV); MN025323.1 (Pigeon, China 12/01/2018, isolate PdCoV/PG/Jiangsu/9-7-9/2018, pigeon-dominant CoV)

Three mutations (CU instead of GG at the end and U to C at position 19,547 of reference strain NC_001451.1, introducing two mismatches in extended duplex) relative reference strain (NC_001451.1)[19]: OP899613.1 (Indigenous chicken, Kenya, 01/31/2017, isolate AvCoV/ck/KE/1922/A376/2017).

One mutation (A to U at position 19,531 of reference strain NC_001451.1, introducing mismatch in extended duplex)[20]: MK574042.1 (*Gallus gallus*, China, 2011, isolate ck/CH/LHB/110615); MK574043.1 (*Gallus gallus*, China, 2011, isolate ck/CH/LHB/110617).

One mutation (U to C at position 19,538 of reference strain NC_001451.1, introducing mismatch in duplex portion with sense = antisense)[21]: **KC008600.1** (Chicken, China, 1985); KX252777.1 (*Gallus gallus*, China, 2007); **MN548286.1** (*Gallus gallus*, United Kingdom, 01/2011); **KY407557.1** (*Gallus gallus*, China, 2014); **KY407558.1** (*Gallus gallus*, China, 2014); **MN197549.1** (Chicken, China, 2016); **MK217374.1** (*Gallus*, China 2016); **MK217375.1** (*Gallus*, China 2016); **MF882923.1** (Chicken, China, 07/2016); **MW815495.1** (Avian, China, 11/03/2020)

One mutation (U to C at position 19,544 of reference strain NC_001451.1, introducing mismatch in duplex portion with sense=antisense)[22]: **MN128087.1** (Chicken, Taiwan, 1998, strain TW2575/98vac, 77th passage, group TW-I [live attenuated vaccine strain]); **KX219797.1** (*Gallus gallus*, China, 01/2002); **KX236001.1** (*Gallus gallus*, China, 2003); **KX252772.1** (*Gallus gallus*, China, 2003); **KX252787.1** (*Gallus gallus*, China, 2003); **KX302866.1** (*Gallus gallus*, China, 2004); **AY646283.1** (IBV, China, 06/05/2004); **KF280267.1** (Chicken, China, 07/12/2004); **KX252778.1** (*Gallus gallus*, China, 2005); **HO848267.1** (*Gallus gallus*, China, 08/05/2005); **DQ646405.1** (IBV, Taiwan, submitted 07/28/2008, isolate TW257598, wild type of MN128087.1); **MF924724.1** (*Gallus gallus*, South Korea, 12/11/2008); **MF924725.1** (*Gallus gallus*, South Korea, 12/11/2008); **KX252780.1** (*Gallus gallus*, China, 2009); **KF6688605.1** (*Gallus gallus*, China, 2009); **KX236007.1**

(*Gallus gallus*, China, 2010); **KX236010.1** (*Gallus gallus*, China 04/2010); **KP118886.1** (*Gallus gallus*, China, 2011); **KX236002.1** (*Gallus gallus*, China, 2011); **KX236003.1** (*Gallus gallus*, China, 2011); **KX247130** (*Gallus gallus*, China, 04/2012); **KX259256.1** (*Gallus gallus*, China, 05/2012); **MT556742.1** (Chicken, USA, 11/2012); **MN987231.1** (Chicken, Egypt, 11/2012); **KX252776.1** (*Gallus gallus*, China, 2013); **KX302870.1** (*Gallus gallus*, China, 2013); **KP343681.1** (*Gallus gallus*, China, 2013); **KF280268.1** (Chicken, China, 06/22/2013); **KF280269.1** (Chicken, China, 06/22/2013); **KF280270.1** (Chicken, China, 06/22/2013); **KX275393.1** (*Gallus gallus*, China, 07/2013); **KX364297.1** (*Gallus gallus*, China, 10/2013); **KX348116.1** (*Gallus gallus*, China, 05/2014); **KX236015.1** (*Gallus gallus*, China, 07/2014); **KU356856.1** (Chicken, China, 09/2014); **KX434789.1** (*Gallus gallus*, China, 2014); **KT852992.1** (*Gallus gallus*, China, 2015, strain: tl/CH/LDt3/03, attenuated); **KX236004.1** (*Gallus gallus*, China, 2015); **KR608272.1** (*Gallus gallus*, China, 05/06/2015); **KX252781.1** (*Gallus gallus*, China, 06/2015); **MN599049.1** (Broiler, US, 07/31/2015); **MT505388.1** (Avian, China, 03/15/2016); **MN987230.1** (Chicken, Egypt, 04/2016); **MN262644.1** (Chicken, Egypt, 2018); **MW896951** (Chicken, China, 04/01/2018); **OK507216.1** (*Gallus gallus*, China, 2020); **OM970248.1** (Chicken, China, 2021, strain CK/CH/GX/202109); **OK423446.1** (*Gallus sp.*, China, 2021); **OQ729967.1** (*Gallus gallus*, Indonesia, 06/06/2021); **OQ729966.1** (*Gallus gallus*, Indonesia, 08/07/2021); **OP823402.1** (IBV, USA, 10/26/2021); **OQ716704.1** (*Gallus gallus*, Indonesia, 11/10/2021); **OP381188.1** (*Gallus gallus*, USA, 10/01/2021); **OQ095389.1** (IBV, USA, 03/03/2022); **OQ716702.1** (*Gallus gallus*, Indonesia, 03/08/2022); **ON036184.1** (*Gallus gallus*, China, 03/18/2022); **ON036185.1** (*Gallus gallus*, China, 03/18/2022)

One mutation (U to C at position 19,547 of reference strain NC_001451.1, introducing mismatch in duplex portion with sense=antisense)[23]: **MK581203.1** (*Gallus gallus*, Poland, 07/1997); **NC_048213.1** (Chicken, India, 2003); **JF699752.1** (Duck, China, 05/2004); JF705860.1 (Duck, China, 05/2004); **JF274479.2** (Chicken, China, 05/07/2007); **EU817497.1** (Chicken, China, 2008); **JF828980.1** (Chicken, China, 2010); **KJ435286.1** (*Gallus gallus*, China, 12/27/2011); **KC013541.1** (Chicken, China, 07/12/2012); **MK937832.1** (*Gallus gallus*, China, 2013); **AY641576.1** (IBV, China, 05/31/2004); **MK937828.1** (*Gallus gallus*, China, 2016); **OP899612.1** (*Gallus domesticus*, Kenya, 01/31/2017); **OM912698.1** (IBV, Mexico, 04/23/2019)

One mutation (G to A at position 19,551 of reference strain NC_001451.1, introducing mismatch in duplex portion with sense=antisense)[24]: **LC634083.1** (*Gallus gallus domesticus*, Japan, 05/28/2021)

One mutation (U to C at position 19,553 of reference strain NC_001451.1, introducing mismatch in duplex portion with sense=antisense)[25]: **GU393336.1** (*Gallus gallus*, USA, 1954); **GU393337.1** (*Gallus gallus*, USA, 1956); **GU393334.1** (*Gallus gallus*, USA, 1960); **GU393338.1** (*Gallus gallus*, USA, 1964); **MK957245.1** (*Gallus gallus*, Brazil, 1988); **JQ977698.1** (*Gallus gallus*, South Korea, 1991); **GQ427175.1** (Turkey, USA, 1994); **MG021194.1** (Chicken, Italy, 1996); **ON419876.1** (*Gallus gallus*, Argentina, 2011); **KU900739.1** (*Gallus gallus*, South Korea, 2003); **KU900740.1** (*Gallus gallus*, South Korea, 2005); **OR268739.1** (*Gallus gallus*, Mexico, 2007); **OR268740.1** (*Gallus gallus*, Mexico, 2007); **OR268741.1** (*Gallus gallus*, Mexico, 2007); **OR268750.1** (*Gallus gallus*, Mexico, 2007); **NC_010800.1** (Turkey, Canada, 08/14/2007); **KU900741.1** (*Gallus gallus*, South Korea,

2009); **MT984585.1** (*Gallus gallus*, Greece, 2010); **KU900743.1** (*Gallus gallus*, South Korea, 2010); **OR050562.1** (Broiler, South Korea, 07/25/2010); **OR050558.1** (IBV, South Korea, 12/10/2010); **MF421320.1** (*Gallus gallus*, Uruguay, 2011); **ON419878.1** (*Gallus gallus*, Uruguay, 2012); **MK618758.1** (Chicken, South Korea, 2012); **ON419877.1** (*Gallus gallus*, Uruguay, 2014); **OR050562.1** (IBV, South Korea, 04/22/2016); **OR050561.1** (Broiler, South Korea, 05/03/2016); **MT984601.1** (*Gallus gallus*, Romania, 2017); **MN199462.1** (*Gallus gallus*, South Korea, 04/05/2017); **OR050554.1** (Broiler, South Korea, 01/30/2019)

Two mutations (U to C and G to A at positions 19,553 and 19,563, respectively, of reference strain NC_001451.1, introducing mismatches in extended duplex)[26]: OR268745.1 (*Gallus gallus*, Mexico, 2019, isolate Mex-1); OR268752.1 (*Gallus gallus*, Mexico, 2019, isolate Mex-3009); OR268742.1 (*Gallus gallus*, Mexico, 2019, isolate Mex-20); OR268752.1 (*Gallus gallus*, Mexico, 2020, isolate Mex-12); OR268749.1 (*Gallus gallus*, Mexico, 2020, isolate Mex-14P); OM912677.1 (*Gallus gallus domesticus*, Mexico/South, 11/25/2020, IBV/ck/MEX/2360/20, serotype: GI-9 [Arkansas type]); OM912678.1 (*Gallus gallus domesticus*, Mexico/South, 11/25/2020, IBV/ck/MEX/2359/20, serotype: GI-9 [Arkansas type]); OM912695.1 (*Gallus gallus domesticus* [Broiler], Mexico/Central, 01/28/2021, IBV/ck/MEX/2523/21, serotype: GI-13 [793B or 4/91-like], [vaccine strain]); OM912694 (*Gallus gallus domesticus*, Mexico/Central, 02/25/2021, IBV/ck/MEX/2562/21, serotype: GI-9 [Arkansas type]); OM912694 (*Gallus gallus domesticus*, Mexico/Central, 02/25/2021, IBV/ck/MEX/2563/21, serotype: GI-9 [Arkansas type]); OM912688.1 (*Gallus gallus domesticus*, Mexico/North, 07/11/2021, IBV/ck/MEX/2742/21, serotype: GI-9 [Arkansas type]); OM912704.1 (*Gallus gallus domesticus*, Mexico/Central, 11/23/2021, IBV/ck/MEX/2930/21, serotype: GI-9 [Arkansas type]); OM912706.1 (*Gallus gallus domesticus*, Mexico/South, 12/14/2021, IBV/ck/Mex/2956/21, serotype: GI-9)

Two mutations (U to C at positions 19,553 and 19,562 of reference strain NC_001451.1, introducing mismatches in extended duplex)[27]: **MN548285.2** (*Gallus gallus*, United Kingdom, 01/2011, strain CR88); **OM912697.1** (Vaccine isolate, Mexico, 04/23/2019, IBV/ck/MEX/1619/19, serotype: CI-13 [793B or 4/91-like])

Two mutations (U to C and a to U at positions 19,553 and 19,557 of reference strain NC_001451.1, introducing mismatches in extended duplex)[28]: **MK618759.1** (Chicken, South Korea, 2012, isolate K04712)

Two mutations (U to C at positions 19,538 and 19,553 of reference strain NC_001451.1, introducing mismatches in extended duplex)[29]: **MN096598.1** (Chicken, China, 2016)

Two mutations (U to C at positions 19,547 and 19,553 of reference strain NC_001451.1, introducing mismatches in duplex portion with sense=antisense)[30]: **KU900744.1** (*Gallus gallus*, South Korea, 2006, QIA-Q43); **MN517817.1** (*Gallus gallus*, China, 2011); **MN509588.1** (*Gallus gallus*, China, 2017)

Two mutations (U to C at positions 19,547 and 19,558 of reference strain NC_001451.1, introducing mismatches in extended duplex)[31]: **MT984594.1** (Gallus gallus, Greece, 2011, strain: D1760/2/2/2011/GR)

Two mutations (U to C and G to A at positions 19,547 and 19,551, respectively, of reference strain NC_001451.1, introducing mismatches in duplex portion with sense=antisense)[32]: **KX364298.1** (*Gallus gallus*, China, 2005, cK/CH/LDL/05II, genotype LX4); **KX348114.1** (*Gallus gallus*, China, 2005, cK/CH/LDL/05III, genotype: LX4)

Two mutations (A to G at position 19,541 and U to C at position 19,544 of reference strain NC_001451.1, introducing mismatch in extended duplex)[33]: **MK581204.1** (*Gallus gallus*, Poland, 1997); **OR268746.1** (*Gallus gallus*, Mexico, 2007, isolate Mex 07-1); **OR268748.1** (*Gallus gallus*, Mexico, 2007, isolate Mex-07-3); **MT984586.1** (*Gallus gallus*, Romania, 2010, strain: D1530/2/3/2010/RO); **MT984587.1** (*Gallus gallus*, Romania, 2010, strain: D1530/4/1/2010/RO); **KX364299.1** (*Gallus gallus*, China, 2010, cK/CH/LSD/101223, genotype: LX4); **KX259250** (*Gallus gallus*, China, 2010, cK/CH/LJS, 10113); **KX219794.1** (*Gallus gallus*, Chicken, 11/2010, cK/CH/LJS,101109, genotype: LX4); **KX302862.1** (*Gallus gallus*, China, 11/2010, cK/CH/LJS/101111, genotype: LX4); **KP118884.1** (*Gallus gallus*, China, 2011, ck/CH/LSD/110851); **KP118885.1** (*Gallus gallus*, China, 2011, ck/CH/LSD/110857); **KP118893.1** (*Gallus gallus*, China, 2011,ck/CH/LSD/110410); **KJ135013.1** (*Gallus gallus*, Ukraine, 10/2011, isolate IBVUkr27-11); **JX195176.1** (Chicken, China, 11/03/2011, ck/CH/LZJ/111113); **KP118883.1** (*Gallus gallus*, China, 2012, ck/CH/LHB/121041); **KP036503.1** (*Gallus gallus*, China, 2012, ck/CH/LHB/121010); **MK581206.1** (*Gallus gallus*, Poland, 12/14/2012); **KP118888.1** (*Gallus gallus*, China, 2013, ck/CH/LLN/130102); **KP118889.1** (*Gallus gallus*, China, 2013, ck/CH/LHB/130575); **KP118890.1** (*Gallus gallus*, China, 2013, ck/CH/LHB/130578); **KP118892.1** (*Gallus gallus*, China, 2013, ck/CH/LLN/13101); **KP036504.1** (*Gallus gallus*, China, 2013, ck/CH/LHB/130630); KP118880.1 (*Gallus gallus*, China, 2013, ck/CH/LHB/130927); **KX236009.1** (*Gallus gallus*, China, 01/2013, cK/CH/LSX/130132, genotype: LX4); **KF377577.1** (IBV, 07/06/2013, 4/91 [vaccine strain], Al-Jallad et al. 2020); **KP118887.1** (*Gallus gallus*, China, 2014, ck/CH/LHB/140532); **KP118881.1** (*Gallus gallus*, China, 2014, ck/CH/LBJ/140413); **KP118882.1** (*Gallus gallus*, China, 2014, ck/CH/LBJ/140402); **MT665806.1** (Chicken, Canada, 2017, IBV/Ck/Can/17-038913); **OQ434268.1** (Chicken, Pakistan, 05/17/2017, Ch/IBV/Pak/AW-2/GI-13/2017, genotype: GI-13); **OR050555.1** (Broiler, South Korea, 12/28/2018, IBV/Korea/193/2018); **MN794188.1** (*Gallus gallus*, China, 2019); **ON044998.1** (*Gallus gallus*, China, 2019, isolate 05/P1-D1/Tr4); **MZ367369.1** (*Gallus gallus*, Belgium, 2019, IBV/chicken/Belgium/4134_001/2019); **OR268744.1** (*Gallus gallus*, Mexico, 2020, isolate Mex-15); **OR050564.1** (Broiler, South Korea, 03/05/2020, IBV/Korea/140/2020); **OM9126879.1** (*Gallus gallus domesticus* (Broiler), Mexico/South, 07/20/2020, IBV/ck/MEX/2752/21, serotype: GI-13 [793B or 4/91-like] [vaccine strain]); **OM912680.1** (*Gallus gallus domesticus* (Broiler), Mexico/South, 11/25/2020, IBV/ck/MEX/2353/20, serotype: GI-13 [793B or 4/91-like] [vaccine strain]); **OM912682.1** (*Gallus gallus domesticus* (Broiler), Mexico/South, 11/25/2020, IBV/ck/MEX/2354/20, serotype: GI-13 [793B or 4/91-like] [vaccine strain]); **OM912692.1** (*Gallus gallus domesticus*, Mexico/South, 04/01/2021, IBV/cx/MEX/2592/21, serotype: GI-13 [793B or 4/91-like] [vaccine strain]); **OP737828.1** (Chicken, China, 04/14/2021, IBV/chicken/Hubei/S1402/2021);

OM912689.1 (*Gallus gallus domesticus*, Mexico/North, 06/06/2021, IBV/ck/MEX/2725/21, serotype: GI-13 [793B or 4/91-like] [vaccine strain]); **OM912690.1** (*Gallus gallus domesticus*, Mexico/North, 06/06/2021, IBV/ck/MEX/2723/21, serotype: GI-13 [793B or 4/91-like] [vaccine strain]); **OM912691.1** (*Gallus gallus domesticus*, Mexico/North, 06/06/2021, IBV/ck/MEX/2721/21, serotype: GI-13 [793B or 4/91-like] [vaccine strain]); **OM912676.1** (*Gallus gallus domesticus*, Mexico/Central, 06/23/2021, IBV/ck/MEX/2731/21, serotype: GI-3 (Holte/Iowa-97]); **OM912686.1** (*Gallus gallus domesticus*, Mexico/South, 07/20/2021, IBV/ck/MEX/2753/21, serotype: GI-13 [793B or 4/91-like] [vaccine strain]); **OM912701.1** (*Gallus gallus domesticus*, Mexico/South, 09/13/2021; IBV/ck/MEX/2826/21, serotype: GI-13 [793B or 4/91-like] [vaccine strain]); **OM912702.1** (*Gallus gallus domesticus*, Mexico/North, 09/27/2021, IBV/ck/MEX/2833/21, serotype: GI-13 [793B or 491-like] [vaccine strain]); **OM912699.1** (*Gallus gallus domesticus*, Mexico,/North, 10/10/2021, IBV/ck/MEX/2818/21, serotype: GI-13 [793B or 4/91-like] [vaccine strain]); **OM912700.1** (*Gallus gallus domesticus*, Mexico,/North, 10/10/2021, IBV/ck/MEX/2819/21, serotype: GI-13 [793B or 4/91-like] [vaccine strain]); **OM912705.1** (*Gallus gallus domesticus*, Mexico/North, 12/07/2021, IBV/ck/Mex/2944/21, serotype: GI-17 [CAV]); **ON044997.1** (*Gallus gallus*, China, 2022, isolate 05/P1-D3/Tr3); **ON044999.1** (*Gallus sp.*, China, 2022, isolate 05/P1-D1/Tr1); **ON081490.1** (*Gallus sp.*, China, 2022, isolate 05/P2-D5/Lu3); **ON081491.1** (*Gallus sp.*, China, 2022, isolate 05/P2-D3/Pr5); **ON081486.1** (*Gallus sp.*, China, 2022, isolate 05/P2-D5/Pr2); **ON081487.1** (*Gallus sp.*, China, 2022, isolate 05/P2-D3/Tr5); **ON081488.1** (*Gallus sp.*, China, 2022, isolate 05/P2-D5/Pr3); **ON081489.1** (*Gallus sp.*, China, 2022, isolate 05/P2-D5/Tr1); **ON081492.1** (*Gallus sp.*, China, 2022, isolate 05/P2-D1/Tr2); **ON081494.1** (*Gallus sp.*, China, 2022, isolate 05/P2-D5/Tr3); **ON081493.1** (*Gallus sp.*, China, 2022, isolate 05/P2-D5/Tr4); **OR824985.1** (*Gallus gallus*, India, 12/18/2022, IBV/India/ck/03/23, genotype: GI-13 [793B or 4/91-like] [vaccine strain]); **OR824986.1** (*Gallus gallus*, India, 12/18/2022, IBV/India/ck/03/23, genotype: GI-13 [793B or 4/91-like] [vaccine strain]); **OR824987.1** (*Gallus gallus*, India, 12/18/2022, IBV/India/ck/03/23, genotype: GI-13 [793B or 4/91-like] [vaccine strain])

Two mutations (A to G at position 19,541 and U to C at position 19,553 of reference strain NC_001451.1, introducing mismatch in extended duplex)[34]: **LN610099.1** (Guinea fowl, France, 2011, gammaCoV/guinea fowl/S/2011)

Two mutations (A to G at position 19,541 and U to C at position 19,563 of reference strain NC_001451.1, introducing mismatch in extended duplex)[35]: **MK778365.1** (*Gallus gallus*, Australia, 1991, Ck/Aus/V18/91, genotype: GIII-1); **MK778364.1** (*Gallus gallus*, Australia, 1992, Ck/Aus/V6/92, genotype: GIII-1)

Two mutations (U to C at positions 19,544 and 19,553 of reference strain NC_001451.1, introducing mismatches in extended duplex)[36]: **GQ504725.1** (Chicken, USA, 1941, strain Mass41 [vaccine strain], Amarasinghe et al 2018); **GU393335.1** (*Gallus gallus*, Netherlands, 1960, serotype: H120 [vaccine strain]); **FJ888351.1** (*Gallus gallus*, Netherlands, 1960, strain H120 [vaccine strain]); **FJ904716.1** (Chicken, USA, 1966, strain: Conn46 1966, serotype: Connecticut [vaccine strain]); **GU393333.1** (*Gallus gallus*, USA, 1971, serotype: FL18288; clusters with Conn 46 vaccine isolates); **FJ904717.1** (Chicken, USA, 1972, strain: Conn46 1972 [vaccine strain], serotype:

Connecticut); **MH021175.1** (*Gallus gallus*, Netherlands, 1979, strain D274 [vaccine strain], genotype: GI-12); **GQ504721.1** (Chicken, USA, 1981, Arkansas vaccine strain); **FJ904718.1** (Chicken, USA, 1983, strain: Conn46 1983, serotype: Connecticut [vaccine strain]); **KR231009.1** (*Gallus gallus*, Belgium, 1984, strain B1648, serotype: Belgian nephropathogenic infectious bronchitis); **MK581200.1** (*Gallus gallus*, Poland, 06/08/1989, strain gammaCoV/Ck/Poland/78/1989); **MK581201.1** (*Gallus gallus*, Poland, 06/08/1989, strain gammaCoV/Ck/Poland/79/1989); **MK581202.1** (*Gallus gallus*, Poland, 06/08/1989, strain gammaCoV/Ck/Poland/80/1989); **GU393331.1** (*Gallus gallus*, USA, 1991, serotype: Cal56B); **FJ904719.1** (Chicken, USA, 1991, strain: Conn46 1991, serotype: Connecticut [vaccine strain]); **GU393332.1** (*Gallus gallus*, USA, 1992, serotype: Delaware 072 [vaccine strain]); **MN566147.1** (Broiler, USA, 1994, [vaccine strain ArkDPI but virulent form also present in group 36], Goraichuk et al. 2020); **FJ904714.1** (Chicken, USA, 1995, strain Cal 1995, serotype: California); **M21515.1** (Chicken embryo kidney cell, Japan, 05/18/1995, strain KB8523, severe respiratory disease, no nephritis or nephrosis); **JX195177.1** (Chicken, China, 1997, strain ck/CH/LDL/97I, substrain P5 [attenuated potential vaccine strain], Zhao et al. 2014); **EU714028.1** (*Gallus gallus*, China, 1997, isolate ZJ971 [non-pathogenic]. Liu X et al. 2013); **GQ504722.1** (Chicken, USA, 1998, strain: Georgia 1998 pass8, virulent); **GQ504723.1** (Chicken, USA, 1998, strain: Georgia 1998 Vaccine from GQ504722.1, avirulent); **ON419888.1** (*Gallus gallus*, Argentina, 2001, isolate AR/01/BA/LDBI-15, recombination of GI-11 and GI-16 lineages); **JX195178.1** (Chicken, China, 2001, strain ck/CH/LDL/97I, substrain P115 [attenuated potential vaccine strain], Zhao et al. 2014); **FJ904715.1** (Chicken, USA, 2003, strain: Cal557 2003, serotype: California); **GQ427173.1** (Turkey, USA, 2003, strain: TCoV/VA-74/03); **KY273667.1** (IBV, Jordan/Massachusetts/USA, 2004, Jordan/Mass/15/2004); **MN711790.1** (Broiler, USA, 10/05/2004, isolate GA/1472/2004, genotype GA08, Var-2 strain); **FJ904713.1** (Chicken, USA, 2006, strain Mass41 2006, serotype: Massachusetts [vaccine strain]); **ON419885.1** (*Gallus gallus*, Argentina, 2006, AR/06/BA/LDBI-20, Var-2 strain); **ON419886.1** (*Gallus gallus*, Argentina, 2006, AR/06/BA/LDBI-22, Var-2 strain); **ON419890.1** (*Gallus gallus*, Argentina, 2006, AR/06/BA/LDBI-19, Var-2 strain); **ON419889.1** (*Gallus gallus*, Argentina, 2006, AR/06/BA/LDBI-21, Var-2 strain); **AY514485.1** (IBV, 04/06/2007, strain Cal99, serotype: California 99); **JF330899.1** (Chicken, China, 2009, ck/CH/LNM/090107 [non-pathogenic] Liu et al., 2013); MF421319.1 (*Gallus gallus*, Uruguay, 2009, UY/09/CA/01); **EU418976.1** (IBV passage 11, USA, 03/06/2009, strain ArkDPI11, virulent, Ammayappan et al. 2009); **EU418975.1** (IBV passage 101, USA, 03/06/2009, strain ArkDPI101, avirulent derived from EU418976.1, Ammayappan et al. 2009); **FJ807652.1** (Embryonated chicken eggs, China, 04/06/2009, strain: H120 [vaccine strain]); **KJ425503.1** (*Gallus gallus*, China, 09/08/2009, ck/CH/LHLJ/090908); KJ425508.1 (Gallus gallus, China, 09/09/2009, ck/CH/LHN/090909); **JF828981.1** (Chicken, China, 2010, ck/CH/LDL/101212 [non-pathogenic], Liu et al. 2013); **MT984588.1** (*Gallus gallus*, Hungary, 2011, strain: D1561/18/2011/HU); **MT984593.1** (*Gallus gallus*, Greece, 2011, strain: D1719/1/2011/GR; **MN548287.1** (*Gallus gallus*, United Kingdom, 01/2011, strain: H120 [vaccine strain]); **MN548288.1** (*Gallus gallus*, United Kingdom, 01/2011, strain italy02, second genotype most prevalent in Italy); **KJ425505.1** (*Gallus gallus*, China, 03/10/2011, ck/CH/LHLJ/110310); **KJ425486.1** (*Gallus gallus*, China, 05/23/2011, ck/CH/LHB/110557); **KJ425487.1** (*Gallus gallus*, China, 05/26/2011, ck/CH/LHB/110526); **KJ435280.1** (*Gallus gallus*, China, 05/29/2011, ck/CH/LSD/110505); **KJ435281.1** (*Gallus gallus*,

China, 05/29/2011, ck/CH/LSD/110529 [derived from flock vaccinated with live attenuated H120]); **KJ435282.1** (*Gallus gallus*, China, 07/26/2011, ck/CH/LSD/110726, [derived from flock vaccinated with live attenuated H120]); **KC506155.1** (Chicken, China, 10/10/2011, ck/CH/LJL111054, originated from a H120-vaccinated chicken, further genomic analysis revealed recombination events between Conn- and Mass-like strains); **KF411040.1** (Chicken, China, 11/22/2011, CK/CH/LLN/111169, originated from multiple recombination events between GI-19 and the Connecticut [Conn] and 4/91 vaccine strains); **KF696629.1** (IBV, China, 08/01/2013, Connecticut vaccine strain); **KJ425488.1** (*Gallus gallus*, China, 08/25/2011, ck/CH/LHB/110825, originated from multiple recombination events between GI-19 and the Connecticut (Conn) and 4/91 vaccine strains); **KJ425485.1** (*Gallus gallus*, China, 09/17/2011, ck/CH/LHB/110931, originated from multiple recombination events between GI-19 and the Connecticut (Conn) and 4/91 vaccine strains, pathogenic to specific pathogen-free chickens); **KJ425506.1** (*Gallus gallus*, China, 10/22/2011, ck/CH/LHLJ/111050, originated from multiple recombination events between IBV strains 4/91 and H120, Zhang et al. 2015); **KJ425489.1** (*Gallus gallus*, China, 11/18/2011, ck/CH/LHB/111172, originated from multiple recombination events between GI-19 and the Connecticut (Conn) and 4/91 vaccine strains, Zhou S et al. 2014); **KJ425491.1** (*Gallus gallus*, China, 12/18/2011, ck/CH/LHB/111268, originated from multiple recombination events between GI-19 and the Connecticut (Conn) and 4/91 vaccine strains, pathogenic to specific pathogen-free chickens, Zhou et al., 2014); **KJ425490.1** (*Gallus gallus*, China, 12/21/2011, ck/CH/LHB/111232, originated from multiple recombination events between GI-19 and the Connecticut (Conn) and 4/91 vaccine strains, pathogenic to specific pathogen-free chickens, Zhou et al. 2014); **KJ435283.1** (*Gallus gallus*, China, 12/19/2011, ck/CH/LSD/111214, originated from multiple recombination events between GI-19 and the Connecticut (Conn) and 4/91 vaccine strains, pathogenic to specific pathogen-free chickens, Zhou et al. 2014); **KJ435284.1** (*Gallus gallus*, China, 12/23/2011, ck/CH/LSD/111241, originated from multiple recombination events between GI-19 and the Connecticut (Conn) and 4/91 vaccine strains, pathogenic to specific pathogen-free chickens, Zhou et al. 2014); **KT203557.1** (Chicken, India, 2012, isolate B17, serotype: Massachusetts, vaccine strain [Poulvac® IB Mass); **MT984596.1** (*Gallus gallus*, Hungary, 2012, strain: D1871/1/1/2012/HU, belongs to the Variant-2 strain [vaccine strain-derived], Al-Jallad et al 2020); **ON419879.1** (*Gallus gallus*, Uruguay, 2012, UY/12/CA/35, GI-16 lineage); **KJ425492.1** (*Gallus gallus*, China, 04/03/2012, ck/CH/LHB/120403, originated from multiple recombination events between GI-19 and the Connecticut (Conn) and 4/91 vaccine strains, pathogenic to specific pathogen-free chickens, Zhou et al. 2014); **KJ425493.1** (*Gallus gallus*, China, 07/21/2012, ck/CH/LHB/120749, originated from multiple recombination events between GI-19 and the Connecticut (Conn) and 4/91 vaccine strains, Han Z et al. 2011); **KJ425509.1** (*Gallus gallus*, China, 10/23/2012, ck/CH/LSD/121059, originated from multiple recombination events between GI-19 and the Connecticut (Conn) and 4/91 vaccine strains, pathogenic to specific pathogen-free chickens, Zhou et al. 2014); **KJ4255494.1** (*Gallus gallus*, China, 10/24/2012, ck/CH/LHB/121024, originated from multiple recombination events between GI-19 and the Connecticut (Conn) and 4/91 vaccine strains, pathogenic to specific pathogen-free chickens, Zhou et al. 2014); **KJ4255495.1** (*Gallus gallus*, China, 10/26/2012, ck/CH/LHB/121040, , originated from multiple recombination events between GI-19 and the Connecticut (Conn) and 4/91 vaccine strains, pathogenic to specific pathogen-free chickens, Zhou et al. 2014); **KJ435285.1**

(*Gallus gallus*, China, 12/28/2012, ck/CH/LSD/121228, originated from multiple recombination events between GI-19 and the Connecticut (Conn) and 4/91 vaccine strains, pathogenic to specific pathogen-free chickens, Zhou et al. 2014); **MG913343.1** (*Gallus gallus*, Brazil, 2013, AvCoV/Gallus gallus/Brazil/sample 22/2013, genotype GI-1); **MG763935.1** (Chicken, India, 2013, isolate IBV/Chicken/Haryana/53/2013, might be a revertant strain originally evolved from the live attenuated vaccine strains used in the region, Jakhesara et al 2018); **KP868573.1** (*Gallus gallus*, China, 2013, CK/CH/LJL/130908, , originated from multiple recombination events between GI-19 and the Connecticut (Conn) and 4/91 vaccine strains, pathogenic to specific pathogen-free chickens, Zhou et al. 2014); **ON419884.1** (*Gallus gallus*, Argentina, 2013, AR/13/BA/A13); **ON419883.1** (*Gallus gallus*, Argentina, 2013, AR/13/BA/A255); **ON419882.1** (*Gallus gallus*, Argentina, 2013, AR/13/CB/A253); **MZ325300.1** (*Gallus gallus*, Cote d'Ivoire, 2013, strain D2334/12/2/13/CI, Var-2-derived strain); **KP780179.1 9** (Chicken, Italy, 05/13/2013, gammaCoV/Ck/Italy/I2022/13, genotype: Q1-like); **KJ425497.1** (*Gallus gallus*, China, 05/24/2013, ck/CH/LHB/130598, originated from multiple recombination events between GI-19 and the Connecticut (Conn) and 4/91 vaccine strains, pathogenic to specific pathogen-free chickens, Zhou et al. 2014); **KJ425496.1** (*Gallus gallus*, China, 05/25/2013, ck/CH/LHB/130573, originated from multiple recombination events between GI-19 and the Connecticut (Conn) and 4/91 vaccine strains, pathogenic to specific pathogen-free chickens); **KJ425498.1** (*Gallus gallus*, China, 06/18/2013, ck/CH/LHB/130642, originated from multiple recombination events between GI-19 and the Connecticut (Conn) and 4/91 vaccine strains, pathogenic to specific pathogen-free chickens, Zhou et al. 2014); **KJ425499.1** (*Gallus gallus*, China, 11/18/2013, ck/CH/LHB/131118); **KJ425500.1** (*Gallus gallus*, China, 11/21/2013, ck/CH/LHB/131132); **KJ425501.1** (*Gallus gallus*, China, 11/26/2013, ck/CH/LHB/131142); **KJ425502.1** (*Gallus gallus*, China, 11/26/2013, ck/CH/LHB/131143); **KJ425507.1** (*Gallus gallus*, China, 12/16/2013, ck/CH/LHJ/131216); **MK937833.1** (*Gallus*, China, 2014, strain ck/CH/LJL/140820); **KY805845.1** (*Gallus gallus*, Egypt, 2014, IBV/Ck/EG/CU/1/2014, GI-1, closely related to vaccine strain H120, Abozeid et al. 2017); **ON419880.1** (*Gallus gallus*, Uruguay, 2014, UY/14/CA/37); **MH779860.1** (*Gallus gallus*, USA, 2014, strain Ark99, serotype: Arkansas [vaccine strain]); **KY588134.1** (IBV, Pakistan, 2015, serotype Massachusetts, Pakistan/Mass/1003/2A/2015, Var-2 strain, Bande et al. 2017); **KY588135.1** (Chicken, Pakistan, 2015 serotype Massachusetts, Pakistan/Mass/1009/13A/2015, Var-2 strain, Bande et al. 2017); **ON419881.1** (*Gallus gallus*, Uruguay, 2015, UY/15/CA/36-1); **KT736031.1** (*Gallus gallus*, China, 04/2015, ck/CH/LDL/150434-I, re-isolation of the H120 vaccine strain introduced into a chicken flock by vaccination); **KT736032.1** (*Gallus gallus*, China, 04/2015, ckCHLDL150434-II); **KY626045.1** (IBV, Brazil, 2016, Massachusetts type vaccine strain, GI-1); **KY047602.1** (*Gallus gallus*, Poland, 02/2016, gammaCoV/Ck/Poland/G052/2016, genotype: GI-23 [Var2-like]); **MK581207.1** (*Gallus gallus*, Poland, 04/07/2016, strain gammaCoV/Ck/Poland/G103/2016); **MN757859.1** (*Gallus gallus* (broiler), Costa Rica, 09/05/2016, isolate CK/CR/1160/12, strain GA 13-like, serotype: GI-17); **MN512434.1** (Chicken, Canada, 2017, isolate IBV/Ck/Can/17-035614); **MN512435.1** (Chicken, Canada, 2017, isolate IBV/Ck/Can/17-036989); **MN512436.1** (Chicken, Canada, 2017, isolate IBV/Ck/Can/18-048192T); **MN512437.1** (Chicken, Canada, 2017, isolate IBV/Ck/Can/18-048430); **MN512438.1** (Chicken, Canada, 2017, isolate IBV/Ck/Can/18-049707); **ON950740.1** (*Gallus gallus*, Canada, 2017, IBV/Ck/Can/2558004); **OM525798.1** (Chicken, Arizona/USA, 2017, isolate FLS/AZ/17, associated with false layer

syndrome); **MK937829.1** (*Gallus*, China, 2017, strain I0306/17); **KY776700.1** (*Gallus gallus*, China, 03/14/2017, gamma CoV/China/I0712/11); **MZ327723.1** (Guinea fowl, France, 05/2017, gCoV/AvCoV/Guineafowl/France/I172562a1/2017); **MZ327724.1** (Guinea fowl, France, 05/2017, gCoV/AvCoV/Guineafowl/France/I172562a2/2017); **OQ434267.1** (Chicken, Pakistan, 05/17/2017, Ch/IBV/Pak/AW-1/GI-1/2017, genotype: GI-1); **KY776701.1** (*Gallus gallus*, China, 2017, gamma CoV/ck/China/IO108/17); **MK581208.1** (*Gallus gallus*, Poland, 07/07/2017, strain gammaCoV/Ck/Poland/G225/2017, Var-2 strain); **ON713866.1** (*Gallus gallus*, Bangladesh, 11/2017, isolate IVL-BD-IB, strain Massachusetts, Var-2 strain); **OR268743.1** (*Gallus gallus*, Mexico, 2018, isolate Mex-Ark1); **MT176421.1** (Chicken, USA, 07/25/2018, IBV/Ck/USA/PA/P1810234-TR, serotype: DMV/1639, Var-2 strain); **MT176422.1** (Chicken, USA, 07/26/2018, IBV/Ck/USA/PA/P1810234-KD, serotype: DMV/1639, Var-2 strain); **MK071267.1** (*Gallus*, Brazil, 10/17/2018, strain H120 [vaccine strain]); **MK878536.1** (Chicken, USA, 01/10/2019, strain DMV/1639); **OM912696.1** (Vaccine isolate, Mexico, 04/23/2019, IBV/ck/MEX/1623/19, serotype: GI-1 [Massachusetts type]); **MK937831.1** (*Gallus*, China, 05/15/2019, strain H120, vaccine strain); **MW436704.1** (*Gallus*, China, 2020, strain IBV/M41/Y185, inactivated vaccine strain); **KR822424.1** (*Gallus*, China, 2020, strain HV80, Var-2 strain); **OP684009.1** (*Gallus*, China, 2020, strain HV80, Var-2 strain); **MZ367367.1** (*Gallus gallus*, Belgium, 2020, IBV/chicken/Belgium/4439_001-iPLTB/2020, 99.95% nucleotide identity to the vaccine strain H120, lineage GI-1); **MZ367368.1** (*Gallus gallus*, Belgium, 2020, IBV/chicken/Belgium/4439_001-PLTB/2020); **MW024789.1** (Gallus gallus [layer], USA, 01/06/2020, strain IA11g2/2000, serotype: GI-17); **PP373115.1** (*Gallus gallus*, Canada, 2021, IBV/Ck/can/21-2455844); **OM912684.1** (*Gallus gallus domesticus* (Broiler), Mexico/South, 04/01/2021, IBV/ck/MEX/2602/21, serotype: GI-1 [Massachusetts type]); **OM912685.1** (*Gallus gallus domesticus* (Broiler), Mexico/North, 04/01/2021, IBV/ck/MEX/2598/21, serotype: GI-1 [Massachusetts type]); **OM912683.1** (*Gallus gallus domesticus* (Broiler), Mexico/South, 07/11/2021, IBV/ck/MEX/2743/21, serotype: GI-1 [Massachusetts type]); **OM912687.1** (*Gallus gallus domesticus*, Mexico/South, 07/15/2021, IBV/ck/MEX/2748/21, serotype: GI-1 [Massachusetts type]), **OM912681.1** (*Gallus gallus domesticus* (Broiler), Mexico/South, 07/20/2021, IBV/ck/MEX/2754/21, serotype: GI-1 [Massachusetts type]); **OM912703.1** (*Gallus gallus domesticus*, Mexico/South, 10/12/2021, IBV/ck/MEX/2860/21, serotype: GI-1 [Mass type]); **LC683779.1** (*Gallus gallus domesticus*, Japan, 02/10/2022, strain: JP/Toyama/2000); **ON350836.1** (*Gallus gallus*, Netherlands, 04/20/2022, strain H120 [vaccine strain]); **ON350837.1** (*Gallus gallus*, China, 04/21/2022, strain r-H120-QX(S) [vaccine strain]); **OR397128.1** (Chicken, Mexico, 04/13/2023, IBV/ck/Mexico/3595-20LM/2023)

Three mutations (U to C at positions 19,544 19,553 and A to G at position 19,558 of reference strain NC_001451.1, introducing mismatch in extended duplex)[37]: **GQ504720.1** (Chicken, USA, 1981, strain Arkansas DPI); **MH779860.1** (*Gallus gallus*, USA, 2014, strain Ark99, serotype: Arkansas); **MH779856.1** (*Gallus gallus*, USA, 2014, isolate ArkGA_P1, strain Ark99, serotype: Arkansas); **MH779857.1** (*Gallus gallus*, USA, 2015, isolate ArkGA_P90, strain Ark99, serotype: Arkansas); **MH779858.1** (*Gallus gallus*, USA, 2016, isolate ArkGA_P40, strain Ark99, serotype: Arkansas)

Two mutations (U to C at positions 19,544 and 19,559 of reference strain NC_001451.1, introducing mismatch in extended duplex)[38]: **KX272465.1** (*Gallus gallus*, Sudan, 10/01/2014, isolate AR-251-15); **MK142676.1** (Chicken, China, 2016, isolate ahysx-1); **MW896953.1** (Chicken, Henan/China, 09/13/2017, isolate 260R-6772); **OP737823.1** (Chicken, China, 03/17/2021, IBV/chicken/Sichuan/C1452/2021); **OP737827.1** (Chicken, China, 05/11/2021, IBV/chicken/Ningxia/N1394/2021); OQ189490.1 (Chicken, China, 06/2021, CK/CH/SX/2106)

Two mutations (U to C at positions 19,544 and 19,547 of reference strain NC_001451.1, introducing mismatches in duplex portion with sense=antisense)[39]: **KX375808.1** (*Gallus gallus*, China, 1999, cK/CH/LHLJ/99I, genotype LX4); **AY319651.1** (IBV, China, 06/10/2003, isolate BJ); **KM213963.1** (*Gallus gallus*, China, 2013, CK/CH/XDC-2/2013); **KX185056.1** (*Gallus gallus*, China, 2014, strain LD3); **KX272465.1** (*Gallus gallus*, China, 05/04/2016, strain LH1); **MT563407.1** (Chicken, China, 04/12/2019, strain SC/SDL/19); **OR180678.1** (Chicken, Dar es Salaam/Bugurini/Tanzania, 11/21/2019, IBV/ck/Tanzania/Dar es Salaam/Bugurini/1995-B01/18, genotype: GI-19)

One mutation (U to A at position 19,553 of reference strain NC_001451.1, introducing mismatch in duplex portion with sense=antisense)[40]: **MW429061.1** (*Gallus*, China, 2020, strain IBV/M41/Y28, M41 is inactivated vaccine strain)

One mutation (U to C at position 19,562 of reference strain NC_001451.1, introducing mismatch in extended duplex)[41]: **KX259253.1** (*Gallus gallus*, China, 2012, strain ck/CH/LHN/120338); **MH878976.1** (*Gallus gallus*, Peru, 02/22/2014, isolate VFAR-047, genotype: GI-16)

Two mutations (A to U and A to C at positions 19,533 and 19,558, respectively, of reference strain NC_001451.1, introducing mismatches in extended duplex)[42]**: MH453802.1** (Red-necked Avocet, Innaminka Regional Reserve/Australia, 2013); **NC_048214.1** (Duck, China, 2014, isolate DK/GD/27/2014); **MK204393.1** (*Anas gracilis* [Grey Teal duck], Victoria, Australia, 2017); **MK204411.1** (*Tadorna tadornoides* [sheduck], Victoria/Australia, 2017)

Three mutations (U to C at positions 19,532, 19,544, and 19,559, respectively, of reference strain NC_001451.1, introducing mismatches in extended duplex)[43]: **OR050547.1** (Chicken [Broiler], South Korea, 04/17/2018, isolate IBV/Korea/61/2018); **OR050559.1** (Chicken [Layer], South Korea, 05/23/2019, isolate IBV/Korea/40/2019); **OR050544.1** (Chicken [Broiler], South Korea, 02/18/2020, isolate IBV/Korea/25/2020); **OR050548.1** (Chicken [Broiler], 04/10/2020, isolate IBV/Korea/63/2020); **OR050549.1** (Chicken [Broiler], 04/17/2020, isolate IBV/Korea/68/2020); **OR050551.1** (Chicken [Broiler], 05/06/2020, isolate IBV/Korea/77/2020); **OR050563.1** (Chicken [Broiler], South Korea, 06/16/2020, isolate IBV/Korea/111/2020); **OQ189491.1** (Chicken, China, 04/2022, CK/CH/SX/2204, genotype: GVI-1)

Three mutations (U to C at positions 19,532, 19,553, and 19,559, respectively, of reference strain NC_001451.1, introducing mismatches in extended duplex)[44]: **OQ749506.1** (Chicken [Farm], China, 07/01/2021, isolate DLSL21)

Three mutations (A to U, U to C, and A to C at positions 19,533, 19553, and 19,558, respectively, of reference strain NC_001451.1, introducing mismatches in extended duplex)[45]: **KT254295.1** (Duck-dominant CoV, China, 2014, isolate DdCoV/DK/Guangdong/F11/2014, GI-11 lineage); **KT254296.1** (Duck-dominant CoV, China, 2014, isolate DdCoV/DK/Guangdong/F40/2014)

Three mutations (U to C at positions 19,544 19,553 and A to C at position 19,557 of reference strain NC_001451.1, introducing mismatches in extended duplex)[46]: **ON419887.1** (*Gallus gallus*, Argentina, 2001, isolate AR/01/BA/LDBI-5, genotype: GI-11)

Three mutations (U to C at position 19,529 and 19,544, and A to G at position 19,541 of reference strain NC_001451.1, introducing mismatches in extended duplex)[47]: **KP662631.1** (*Gallus gallus*, South Africa, 06/01/2011, isolate ck/ZA/3665/11, serotype: QX-like)

Three mutations (C to G at position 19,537, A to G at position 19,541, U to C at position 19,544, of reference strain NC_001451.1, introducing mismatches in extended duplex)[48]: **KP118891.1** (*Gallus gallus*, China, 2011, strain ck/CH/LHLJ/111246; genotype: GI-13)

Three mutations (U to C at position 19,534, A to G at position 19,541, U to C at position 19,544, of reference strain NC_001451.1, introducing mismatches in extended duplex)[49]: **KP036502.1** (*Gallus gallus*, China, 2014, ck/CH/LHLJ/140906)

Three mutations (U to C at positions 19,553, and 19,559, and G to A at position 19,563, of reference strain NC_001451.1, introducing mismatches in extended duplex)[50]: **OM912708.1** (*Gallus gallus domesticus* [Broiler], Mexico/Central, 12/15/2021), IBV/ck/MEX/2961/21, serotype: GI-9 [Arkansas type])

Three mutations (A to G at position 19,530, A to U at position 19,531, and U to C at position 19,559, of reference strain NC_001451.1, introducing mismatches in extended duplex)[51]: **MW896949.1** (Chicken, Jiangsu/China, 09/09/2017, isolate 71-109646)

Three mutations (U to C at positions 19,532 and 19,544, and U to A at position 19,562, of reference strain NC_001451.1, introducing mismatches in extended duplex)[52]: **MZ325299.1** (*Gallus gallus*, Cote d'Ivoire, 2013, strain D2334/11/2/3/CI)

Three mutations (A to G at position 19,541, and U to C at positions 19,544 and 19,562, of reference strain NC_001451.1, introducing mismatches in extended duplex)[53]: **MT367412.1** (*Meleagris gallopavo*/Turkey, Poland, 06/21/2016, gammaCoV/Tk/ Poland/G160/2016)

Three mutations (U to C at positions 19,544 and 19,559, and A to G at position 19,550, of reference strain NC_001451.1, introducing mismatches in extended duplex)[54]: **GQ427176.1** (Turkey, USA, 1998, TCoV/TX-1038/98); **GQ427174.1** (Turkey, USA, 2001, TCoV/TC-GL/01); **EU022525.1** (Turkey, Indiana/USA, 07/08/2007, TCoV-540)

Three mutations (U to C at positions 19,544 and 19,547, and A to G at position 19,550, of reference strain NC_001451.1, introducing mismatches in extended duplex)[55]: **AY338732.1** (Chicken, China, 01/23/2003, strain LX4, nephropathogenic)

One deletion and 2 mutations (deletion of A at position 19,541, and U to C mutations at positions 19,544 and 19,547, of reference strain NC_001451.1, introducing mismatches in extended duplex)[56]: **EU526388.1** (Chicken, China, strain A2, 02/20/2008, serotype: 4/91-like, a predominant strain in China)

Four mutations (U to C at position 19,529, C to U at position 19,542, U to A at position 19,544, and G to A at position 19,563 of reference strain NC_001451.1, introducing mismatches in extended duplex)[57]: **MZ325298.1** (*Gallus gallus*, Ghana, 2013, strain D2328/15/3/13/GH, genotype: GI-19 [QX-like])

Four mutations (A to U and C at positions 19,533 and 19,558, respectively, and U to C at positions 19,544 and 19,562, of reference strain NC_001451.1, introducing mismatches in extended duplex)[58]: **MT993597.1** (Duck CoV 2714 [*Arenaria interpres*, ruddy turnstone], Tasmania/Australia, 2015, isolate MW11-10)

Five mutations (A to C at position 19,531, U to A at position 19,532, A to G at positions 19,541 and 19,557, and G to A at position 19,556, of reference strain NC_001451.1, introducing mismatches in extended duplex)[59]: **NC_046965.1** (Canada goose CoV [*Branta canadiensis*], Canada, 08/2017, strain Cambridge_Bay_2017); **OR346994.1** (Swan goose CoV, China, 06/2022, isolate DWY40)

Six mutations (A to C at position 19,531, U to A at position 19,532, A to G at positions 19,541 and 19,557, U to C at position 19,544, and G to A at position 19,556, of reference strain NC_001451.1, introducing mismatches in extended duplex)[60]: **MW588092.1** (Mute swan feces CoV, United Kingdom, 01/29/2021, strain Abbottsbury/A/2016)
